# 

**Published:** 1877-04

**Authors:** 


### CONTENTS.

COMMUNICATIONS.

Address . . . . . . . . . . 141
Neuralgia . . . . . . . . . 150
Transactions of the Mississippi Dental Association 154
I Rise to Explain . . . . . . . . 168
A Singular Case in Practice . . . . . . 170

SELECTIONS.

Dental Education . . . . . . . . 174

EDITORIAL.

Ohio Dental College. . . . . . . . 177
New York State Dental Society . . . . 178
Illinois State Dental Society . . . . . 178
Merrimack Valley Dental Society . . . . 179
Eastern Indiana Dental Association . . . . 180
Dental Societies . . . . . . . . 181
The Diamond Disks . . . . . . . 182
Saliva Extractor . . . . . . . . 182
Dental Office and Laboratory . . . . . 182
Commencement . . . . . . . . 183
Commencement . . . . . . . . . 183
Vick's Flower and Vegetable Garden . . . 184

